# Value of transluminal attenuation gradient of stress CCTA for diagnosis of haemodynamically significant coronary artery stenosis using wide-area detector CT inpatients with coronary artery disease: comparison with stress perfusion CMR

**DOI:** 10.5830/CVJA-2017-026

**Published:** 2018

**Authors:** Yeong Kim Hee, Seok Yong Hwan, Kang Eun-Young, Ju Kim Eung, Kyoung Seo Bo

**Affiliations:** Department of Radiology, Kangnam Sacred Heart Hospital,Hallym University College of Medicine, Seoul, Korea; Department of Radiology, Korea University Guro Hospital,Korea University College of Medicine, Seoul, Korea; Department of Radiology, Korea University Guro Hospital,Korea University College of Medicine, Seoul, Korea; Division of Cardiology, Cardiovascular Centre, KoreaUniversity Guro Hospital, Korea University College ofMedicine, Seoul, Korea; Department of Radiology, Korea University Ansan Hospital,Korea University College of Medicine, Seoul, Korea

**Keywords:** coronary artery disease, transluminal attenuation gradient, computerised tomography, magnetic resonance imaging

## Abstract

**Introduction:**

This study aimed to evaluate the value oftransluminal attenuation gradient (TAG) of stress coronarycomputed tomography angiography (CCTA), using a wideareadetector CT in patients with coronary artery disease,compared to stress perfusion cardiac magnetic resonance(CMR) imaging.

**Methods:**

This prospective study from May 2012 to January2015 included 21 patients with moderate coronary stenosison invasive coronary angiography. All patients underwentadenosine stress single-shot CCTA with a rest CCTA scanusing a wide-area detector CT. Coronary artery stenosis wasevaluated on both stress and rest CCTA images, and TAG wasmanually obtained for all vessels. Stress perfusion CMR wasused as a reference standard. A TAG cut-off value of –15.1HU/10 mm was applied for diagnosing haemodynamicallysignificant stenosis. The diagnostic accuracies of TAG andCMR were estimated and compared.

**Results:**

TAG of stress CCTA in all coronary arteries hada sensitivity, specificity, and positive and negative predictivevalues of 90.5, 90.0, 86.4 and 93.1%, respectively.Corresponding values for TAG of rest CCTA in all coronaryarteries were 42.9, 83.3, 64.3 and 67.6%, respectively, whereasthose for TAG of coronary arteries with moderate stenosison stress CCTA were 93.3, 100, 100 and 92.3%, respectively.Mean effective radiation doses for stress and rest CCTA were10.6 ± 2.6 mSv and 2.3 ± 1.3 mSv, respectively.

**Conclusions:**

TAG of CCTA provided high diagnostic accuracyfor detecting haemodynamically significant coronaryartery stenosis. TAG of stress CCTA was more diagnosticallyaccurate, especially in coronary arteries with moderatestenosis.

## Introduction

Coronary computed tomography angiography (CCTA) isincreasingly used as a non-invasive diagnostic imaging toolfor the detection and exclusion of coronary artery disease(CAD).^1^,^2^ However, a well-recognised limitation of CCTA isits moderate ability to assess the haemodynamic significanceof a given coronary stenosis.^3^ Other modalities, such as singlephotonemission computerised tomography (SPECT), cardiacmagnetic resonance (CMR) imaging, invasive fractional flowreserve (FFR), CT-derived computed fractional flow reserve(CT-FFR), or CT myocardial perfusion (CTP) can predicthaemodynamically significant coronary artery stenosis ormyocardial ischaemia. However, CTP imaging may requireadditional iodinated contrast and radiation exposure,^4^,^5^ and theanalysis of CT-FFR data requires a large amount of time ona parallel supercomputer,^6^ even though these modalities weredeveloped in an attempt to improve the diagnostic accuracy ofCCTA.

Recently, the transluminal attenuation gradient (TAG), defined as the contrast opacification gradient along the length of a coronary artery on CCTA, has been suggested as a tool for detecting haemodynamically significant coronary artery stenosis. TAG combines anatomical and functional information to enable appropriate therapeutic decisions regarding CAD. Preliminary data suggest that TAG provides additional functional information to CCTA.^7^,^8^ This method may represent a simple and useful test to differentiate individuals who will or will not benefit from revascularisation.

Choi et al.^8^ reported that TAG could provide information about the functional significance of coronary artery stenosis. However, that study was performed with a 64-slice multi-detector row scanner. Although a subsequent study was performed with a 320-detector row CT scanner, rest CCTA was performed without stress CCTA.^7^ A further study performed both stress and rest CCTA scans with a 320-detector row CT scanner,^9^ but the rest CCTA scan was followed by a stress CCTA scan. All of these studies used invasive FFR as the reference standard.

Invasive FFR is a well-established and highly accurate method for assessing the functional significance of coronary artery stenosis; however, it is limited by its invasive nature. Stress perfusion CMR is a well-established and highly accurate non-invasive method used to assess the functional significance of coronary artery stenosis. Therefore, we designed a study protocol based on CCTA using a wide detector in which a stress scan was followed by a rest scan, and stress perfusion CMR was used as a reference standard.

The aim of this study was to determine whether TAG could be valid for detecting haemodynamically significant coronary artery stenosis using wide-area detector CT, compared to the reference standard of stress perfusion CMR as a reference standard.

## Methods

This prospective study was approved by the institutional review board. Informed consent was obtained from all subjects prior to examination. From May 2012 to January 2015, all patients with moderate coronary artery stenosis (50–70%) detected on invasive coronary angiography (ICA), who were required to undergo haemodynamic significance testing were enrolled, and underwent adenosine stress CCTA and stress perfusion CMR. Exclusion criteria included a history of coronary artery bypass graft surgery or other cardiac surgery, myocardial infarction (MI) or heart failure, atrial fibrillation, second- or third-degree atrioventricular block, impaired renal function, symptomatic asthma, pregnancy or any contra-indications to iodinated contrast agents, or other any MR imaging contra-indication.

## Stress CCTA protocol

All patients were scanned on a wide-area detector CT scanner (Aquilion ONE, Toshiba Medical System, Otawara, Japan) with 320-detector rows (each 0.5-mm wide) and a gantry rotation time of 350 ms. The entire heart was imaged in a single heart beat with a maximum of 16-cm coverage in the Z direction.

**Fig. 1:**
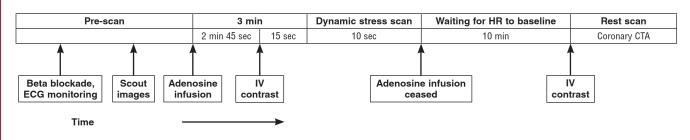
CCTA protocol. After a 3-min intravenous adenosine infusion, contrast-enhanced stress CCTA was acquired, followed by a rest CCTA after 10 minutes. CCTA = coronary computed tomography angiography.

After intravenous adenosine infusion (140 μg/kg/min for three minutes; Denosin injection 90 mg/30 ml; BC World Pharm Co, Ltd, Seoul, Korea), stress CCTA was performed using the biphasic injection method (Fig. 1). A 60-ml bolus of iodinate contrast (lobitridol, Xenetics 350; Guerbet, Paris, France) was injected intravenously, followed by a 50-ml saline chaser at a flow rate of 5 ml/s. To identify the optimal phase of contrast enhancement for adenosine stress CCTA, we performed a 10-second dynamic scan 15 seconds after initiating contrast injection.10 All scans used prospective electrocardiogram (ECG) gating that covered phases 30–50% of the R-R interval.

Rest CCTA was performed 10 min after adenosine stress CCTA. The rest scan was acquired during the injection of 50 ml of iodinate contrast, followed by 50 ml of saline at a flow rate of 5.0 ml/s. The phase window was set at 30–50% of the R-R interval in patients with a heart rate (HR) ≥ 75 beats per minute (bpm), and 65–85% of the R-R interval in patients with a HR < 75 bpm. For most cases, prospective ECG gating covering 65–85% of the R-R interval was used.

## Stress perfusion CMR protocol

CMR was performed using a 3.0-T unit (Magnetom Skyra; Siemens, Erlangen, Germany) with an 18-channel body coil. The imaging protocol consisted of three parts: ciné imaging for ventricular volume and function; first-pass contrast-enhanced myocardial perfusion imaging during adenosine-induced stress and under resting conditions; and myocardial delayed enhancement imaging.

For the perfusion study, adenosine was injected as described for the CCTA protocol, after which 0.05 mmol/kg of gadoliniumbased contrast material (gadoterate meglumine, Dotarem; Guerbet, Villepinte, France) was injected intravenously at an injection rate of 3 ml/s, followed by a 25-ml saline flush. Firstpass stress myocardial perfusion imaging of three short-axis imaging planes positioned in the base, mid and apical myocardial segments of the left ventricle was performed using a saturationrecovery turbo-fast low-angle shot (FLASH) gradient echo sequence. Fifteen minutes after stress perfusion imaging, rest perfusion images were acquired after a second bolus of 0.1 mmol/kg gadolinium-based contrast was injected.

## Analysis of CCTA and CMR imaging

Adenosine stress CCTA data and stress perfusion CMR images were reviewed by two experienced readers (six and 16 years of experience with CCTA and CMR), blinded to the ICA results. Three major coronary arteries per patient were evaluated. TAG was manually obtained for each vessel using an image postprocessing workstation (Vitrea 6.4; Vital Images, A Toshiba Medical Systems Group, Minnetonka, MN, USA), following the method described by Wong et al.^7^

The centre line was determined for each major coronary artery, and cross-sectional images perpendicular to the vessel centre line were subsequently reconstructed. The region of interest (ROI) contour (size = 1 mm2) was positioned in the centre of each crosssectional image. The mean luminal radiological attenuation (in Hounsfield units, HU) was measured at 5-mm intervals from the ostium to a distal level where the vessel cross-sectional area fell below 2.0 mm2.

TAG was determined from the change in HU per 10-mm length of the coronary artery and defined as the linear regression coefficient between intraluminal radiological attenuation (HU) and distance from the ostium (mm).^7^,^8^ A TAG cut-off value of –15.1 HU/10 mm was defined as significant, as previously described.^7^ The TAGs of all coronary arteries and of coronary arteries with moderate stenosis were calculated on stress and rest CCTA scans, and the TAG results were compared with perfusion defects detected on CMR images.

## Radiation dose estimation of coronary CTA

The effective radiation dose of CCTA was calculated by multiplying the dose–length product (DLP) by the conservative constant k (k = 0.014 mSv/mGy/cm), according to standard methodology outlined in the European guidelines on quality criteria for computed tomography.^11^

## Statistical analysis

All continuous variables are expressed as means ± standard deviations, whereas categorical data are expressed as percentages. The diagnostic accuracy of CT-TAG for the detection of perfusion defects was assessed using CMR as the reference standard.

The sensitivity, specificity, positive predictive value (PPV) and negative predictive value (NPV) were calculated and 95% confidence intervals (CI) were reported for each parameter, which were bias–adjusted by bootstrap resampling with replacement 200 times from the sample. Calculations were performed on both an individual coronary vessel and individual patient basis. All reported diagnostic values werThe sensitivity, specificity, positive predictive value (PPV) and negative predictive value (NPV) were calculated and 95% confidence intervals (CI) were reported for each parameter, which were bias-adjusted by bootstrap resampling with replacement 200 times from the sample. Calculations were performed on both an individual ce based on consensus between the two observers. A p-value < 0.05 was considered to indicate statistical significance. Statistical analysis was performed using MedCalc software (Mariakerke, Bergium).

## Results

A total of 21 patients were included in this study. Of these, two declined to undergo stress perfusion CMR after stress CCTA because of chest discomfort during the CCTA scan, and another two patients were excluded because of poor CT image quality that was unsuitable for analysis. Three major coronary arteries per patient were evaluated; therefore, a total of 17 patients (mean age: 60.2 ± 9.5 years; 52.9% men) and 51 coronary arteries successfully underwent the evaluation, with good diagnostic image quality. Patient characteristics are summarised in Table 1.

**Table 1:** Baseline characteristics in 17 patients

Age (years)	60.2 ± 9.5
Men/women	9/8
BMI (kg/m2)	25.0 ± 4.9
Family history of CAD, n (%)	3 (17.6)
Diabetes, n (%)	5 (29.4)
Hypertension, n (%)	8 (47.1)
Hypercholesterolaemia, n (%)	6 (35.3)
Current smoker, n (S%)	4 (23.5)

Data are presented as the mean ± standard deviation or frequency (%). BMI = body mass index; CAD = coronary artery disease.

The mean estimated radiation effective doses for stress and rest CCTA were 10.6 ± 2.6 and 2.3 ± 1.3 mSv, respectively. The mean HR values were 85.5 ± 25.4 bpm during stress and 64.6 ± 10.5 bpm at rest (p = 0.018). The CT scan parameters are summarised in Table 2.

**Table 2:** Stress and rest CCTA imaging parameters

Age (years)	60.2 ± 9.5
Men/women	9/8
BMI (kg/m2)	25.0 ± 4.9
Family history of CAD, n (%)	3 (17.6)
Diabetes, n (%)	5 (29.4)
Hypertension, n (%)	8 (47.1)
Hypercholesterolaemia, n (%)	6 (35.3)
Current smoker, n (%)	4 (23.5)

Data are presented as the mean ± standard deviation or frequency (%). CCTA = coronary computed tomography angiography, bpm = beats per minute.

## Accuracy of TAG compared with stress perfusion CMR

On ICA, all patients had at least one segment containing ≥ 50% stenosis. Six patients (6/17; 35.3%) had single-vessel disease, seven (7/17; 41.2%) had two-vessel disease, and four (4/17; 23.5%) had three-vessel disease. Overall, five vessels (5/51; 9.8%) in three patients (3/17; 17.6%) were found to have at least one segment with ≥ 70% stenosis; these patients had three-vessel disease.

Of the 51 vessels, 19 (19/51; 37.3%) were classified as having mild stenosis [three left anterior descending arteries (LAD), seven left circumflex arteries (LCX) and nine right coronary arteries (RCA)], 27 (27/51; 52.9%) were classified as having moderate stenosis (13 LAD, eight LCX and six RCA), and five vessels (5/51; 9.8%) were classified as having severe stenosis (one LAD, two LCX and two RCA). The mean degree of coronary stenosis in all coronary arteries was 55.1 ± 17.3%.

Regarding TAG of stress CCTA, 22 vessels (22/51; 43.1%) were classified as functionally significant stenosis with a TAG less than –15.1 HU/10 mm, whereas 29 vessels (29/51; 56.9%) had a TAG greater than –15.1 HU/10 mm, indicating functionally non-significant stenosis.

In a patient-based analysis, the sensitivity, specificity, PPV and NPV for TAG of stress CCTA in all patients were 90.0% (9/10; 95% CI, 55.5–98.3%), 71.4% (5/7; 95% CI, 29.3–95.5%), 81.8% (9/11; 95% CI, 48.2–97.2%) and 83.3% (5/6; 95% CI, 36.1–97.2%), respectively. The corresponding values for TAG of rest CCTA in all patients were 66.7% (6/9; 95% CI, 30.1–92.1%), 57.1% (4/7; 95% CI, 18.8–89.6%), 66.7% (6/9; 95% CI, 30.1– 92.1%) and 57.1% (4/7; 95% CI, 18.8–89.6%), respectively. The diagnostic accuracy of the per-vessel analysis was slightly higher than that of the per-patient analysis (Table 3).

**Table 3:** Overall sensitivity, specificity, PPV and NPV of TAG of the coronary arteries with moderate stenosis, all coronary arteries and per-patient analysis on stress and rest CCTA scans

	*Stress CCTA*			*Rest CCTA*		
	*Moderate stenosis (n = 27)*	*All vessels (n = 51)*	*Per patient (n = 17)*	*Moderate stenosis (n = 27)*	*All vessels (n = 51)*	*Per patient (n= 17)*
Sensitivity	93.3 (14/15)	90.5 (19/21)	90 (9/10)	46.7 (7/15)	42.9 (9/21)	66.7 (6/9)
Specificity	100.0 (12/12)	90.0 (27/30)	71.4 (5/7)	83.3 (10/12)	83.3 (25/30)	57.1 (4/7)
PPV	100.0 (14/14)	86.4 (19/22)	81.8 (9/11)	77.8 (7/9)	64.3 (9/14)	66.7 (6/9)
NPV	92.3 (12/13)	93.1 (27/29)	83.3 (5/6)	55.6 (10/18)	67.6 (25/37)	57.1 (4/7)

All data are percentages. The absolute numbers used to calculate the percentages are in parentheses.

PPV = positive predictive value, NPV = negative predictive value, TAG = transluminal attenuation gradient, CCTA = coronary computed tomography angiography.

In a vessel-based analysis, the sensitivity, specificity, PPV and NPV for TAG of stress CCTA in all coronary arteries were 90.5% (19/21; 95% CI, 69.6–98.5%), 90.0% (27/30; 95% CI, 73.4–97.8%), 86.4% (19/22; 95% CI, 65.1–96.9%) and 93.1% (27/29; 95% CI, 77.2–99.0%), respectively. The corresponding values for TAG of rest CCTA in all coronary arteries were 42.9% (9/21; 95% CI, 21.9–65.9%), 83.3% (25/30; 95% CI, 65.3–94.3%), 64.3% (9/14; 95% CI, 35.2–87.1%) and 67.6% (25/37; 95% CI, 49.5–82.6%), respectively.

In five (5/51; 9.8%) vessels (one LAD, two LCX and two RCA), TAG of stress CCTA was not consistent with the findings of stress perfusion CMR. Because two RCAs were hypoplastic and one LCX exhibited diffuse atherosclerotic changes that could not influence the HU gradient, the TAG values of these coronary arteries met the criteria for functionally significant stenosis, but no perfusion defects were observed on stress perfusion CMR. On the other hand, one LCX had stenosis in the far distal portion of the coronary artery, and one LAD had stenosis of the coronary ostium. TAG values of these vessels met the criteria of functionally non-significant stenosis, but CMR images acquired during stress and at rest showed a complete, reversible sub-endocardial perfusion defect.

In coronary arteries with moderate stenosis on ICA, the sensitivity, specificity, PPV and NPV for TAG of stress CCTA were 93.3% (14/15; 95% CI, 68.0–98.9%), 100% (12/12; 95% CI, 73.4–100%), 100% (14/14; 95% CI, 76.7–100%) and 92.3% (12/13; 95% CI, 63.9–98.7%), respectively. The sensitivity, specificity and PPV for TAG of coronary arteries with moderate stenosis were higher than the corresponding values for all vessels on both stress and rest CCTA (Table 3).

## Discussion

**Fig. 2:**
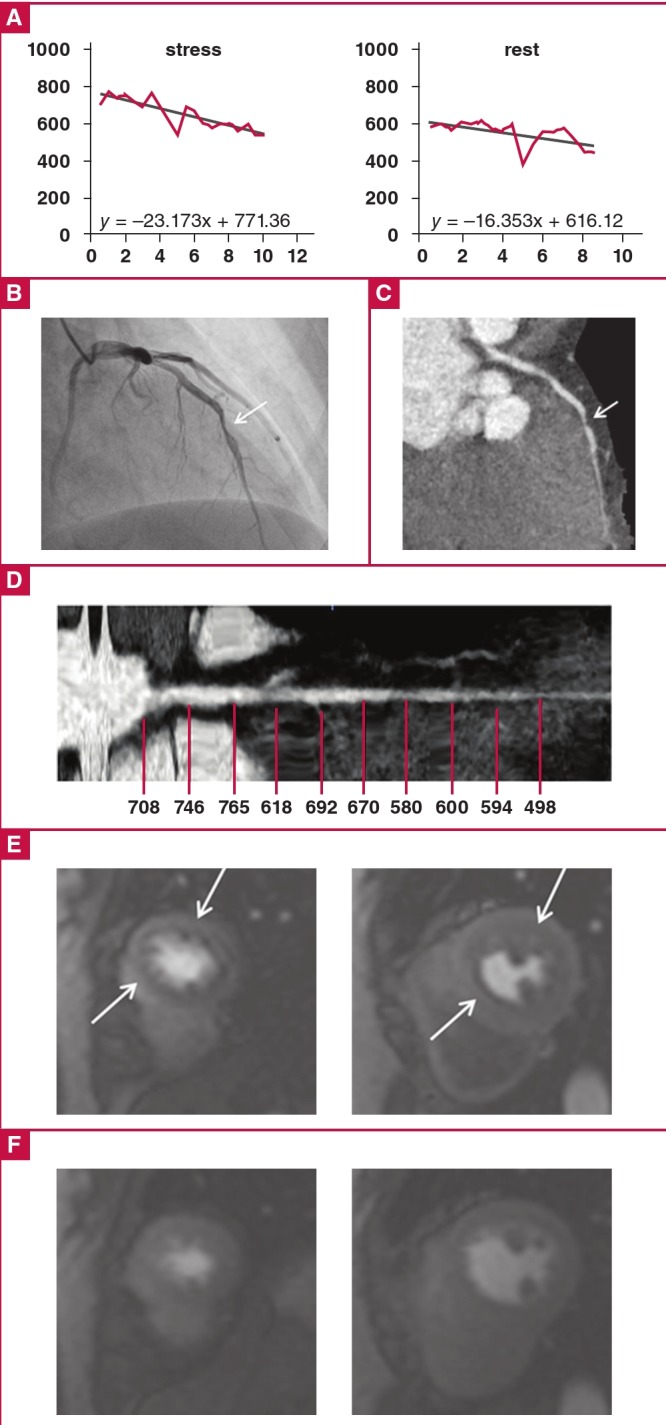
A 47-year-old woman with chest pain. (A) TAG of the LAD was –23 HU/10 mm on stress CCTA and –16 HU/10 mm on rest CCTA. (B) Invasive coronary angiography, and (C) curved multi-planar reformatted images of stress CCTA show significant stenosis in the mid-LAD. (D) Axial multi-planar reformatted image shows corresponding luminal attenuation of CCTA. (E) CMR imaging with stress perfusion imaging shows low signal intensity, indicating a subendocardial perfusion defect in the anterior septum and anterior wall at the mid-ventricular level (LAD territory). (F) No low-signal intensity lesion was visible with rest perfusion imaging. TAG = transluminal attenuation gradient, LAD = left anterior descending artery, CCTA = coronary computed tomography angiography, CMR = cardiac magnetic resonance.

Our data show that the TAG of stress CCTA for the detection of haemodynamically significant coronary artery stenosis yielded an excellent diagnostic performance, and higher accuracy was observed in the coronary arteries with moderate stenosis than for all vessels (Fig. 2). This indicates that TAG could facilitate decisions regarding which coronary arteries would benefit from revascularisation in patients with CAD.

It is widely known that anatomical measures of stenosis are not good predictors of functionally significant stenosis. The high sensitivity of CCTA has been validated in prospective multi-centre studies, although specificity is a known limitation of CCTA.^12^ Invasive FFR was shown to reduce the rate of the composite end-point of death, non-fatal MI and repeat revascularisation in patients with multi-vessel coronary artery disease in the FAME [Fractional Flow Reserve (FFR) versus Angiography in Multivessel Evaluation)] study.^13^ However, FFR is limited by its invasive nature, and non-invasive approaches such as CT-FFR, SPECT and CMR are needed.

More recently, CT-FFR was described in the DISCOVERFLOW (Diagnosis of Ischemia-Causing Stenoses Obtained Via Noninvasive Fractional Flow Reserve) study.^6^ Although the study results have been encouraging, this technique requires much time as well as analysis on a parallel supercomputer. By contrast, TAG can be applied to any CCTA study in daily practice. This technique does not require any modification of CCTA protocols and involves a relatively simple analysis without the need for complicated software, therefore suggesting the possibility of a non-invasive functional assessment of coronary stenosis.^7^

Contrast opacification on single-shot CCTA with wide-area detector CT is homogenous along the length of a normal coronary artery, but exhibits a linear drop-off in luminal HU along the length of the artery in the presence of haemodynamically significant coronary artery stenosis.^14^ Choi et al. reported a low sensitivity but high specificity for TAG measured in 64-detector row CCTA that, when added to CCTA percentage stenosis information, significantly increased the area under the receiver operating characteristic curve for the detection of an abnormal invasive FFR < 0.8.15 However, an earlier study of TAG was performed using a 64-slice multi-detector row scanner,^8^,^15^ which had a limitation of temporal heterogeneity in coronary artery opacification.

Wong et al.^7^ reported that TAG independently predicted FFR < 0.8 and increased both the sensitivity and specificity of information on CCTA percentage stenosis. That study used a 320-detector row CT scanner, but performed rest CCTA without stress CCTA. Thereafter, another study compared the diagnostic accuracy of combined CTP and TAG320.^9^ Both stress and rest CCTA were performed using 320-detector row CT scanner, but an initial rest CCTA scan was followed by a stress CCTA scan. As a result, cross-contamination of contrast during the second acquisition (stress CCTA) might have led to false negatives.

We used a CCTA protocol in which the stress phase is followed by the rest phase during a single examination, which differs from the protocols used in previous studies. Performing a stress phase acquisition first could allow a ‘clean’ acquisition, thus optimising the detection of haemodynamically significant coronary artery stenosis by avoiding contrast contamination.^16^ This protocol is suitable for patients with an intermediate to high pre-test probability of CAD, patients with high calcium scores (> 400 mg/dl), and patients with known CAD. We used a CT protocol in which the stress phase was followed by the rest phase because our patients had exhibited moderate coronary artery stenosis during ICA.

Interpretation of TAG may be limited by multiple heartbeat acquisition algorithms and coronary calcification. The use of a wide-area detector allows a longitudinal axis of 16 cm, which in most instances enables the entire heart volume to be imaged in a single gantry rotation with a short breath-hold time. This is ideal for TAG functional assessments of coronary arterial stenosis because this modality would enable non-invasive quantitative assessment of coronary contrast changes with temporal uniformity,^7^,^14^ and eliminate step registration artifacts.

Radiation dose is a major issue concerning the clinical application of stress CCTA. To determine the optimal enhancement time, a 10-s stress CCTA scan was performed, causing a relative increase in the radiation dose of our protocol (10.6 ± 2.6 mSv) relative to the doses of previous studies. The radiation dose may decrease with a static scan or dynamic stress CCTA with a shorter scan duration. Although dynamic scanning results in higher radiation doses, its advantages includes the ability to determine the optimal enhancement time or to generate dynamic data sets for the visual analysis of serial dynamic images. Further studies are needed to reduce the radiation doses from dynamic CT scans before implementing widespread use. To our knowledge, we have compared for the first time the diagnostic accuracy of TAG of stress CCTA using a wide-area detector CT with that of stress perfusion CMR as a reference standard. In many studies, invasive FFR is becoming more widely accepted and is selected as the reference standard, but FFR has disadvantages such as its invasive nature, the associated radiation exposure, and high costs.^17^

Stress perfusion CMR has been established as a non-invasive diagnostic modality with a high diagnostic accuracy for inducible perfusion defects. This modality has the advantage of no radiation exposure or attenuation artifacts. The diagnostic accuracy of stress perfusion CMR is significantly greater than that of SPECT,^18^ and a qualitative visual analysis of CMR versus FFR identified an excellent diagnostic accuracy for the detection of functionally significant CAD, using a FFR cut-off value < 0.75 for discriminating haemodynamically significant from non-significant stenosis.^19^,^20^ CMR has become an important non-invasive diagnostic modality for the clinical work-up of patients with significant coronary artery stenosis.

In five (9%) coronary arteries (one LAD, two LCX and two RCA), the TAGs of stress CCTA were not consistent with the findings of stress perfusion CMR. Two RCAs were hypoplastic, and one LCX exhibited diffuse atherosclerotic changes. On the TAGs of these vessels, the transverse graph axis, which represents the distance from the ostium to the distal coronary artery, was relatively short, and the diameters of the coronary arteries were small, and TAG indicated a false positive. On the other hand, the TAG of one LCX and one LAD indicated a false negative. The LCX had stenosis of the far distal portion of the coronary artery, and the LAD had stenosis of the coronary ostium. The stenotic portion of the coronary artery was the beginning or end-point on the transverse graph axis, and therefore the intraluminal attenuation gradient of these vessels was not affected by HU in the stenotic portions of the coronary arteries.

## Limitations

This study has some limitations. First, this was a single-centrestudy with a small sample of patients who underwent ICA.Larger, multi-centre studies are needed to provide furtherfunctional information and confirmation, using stress perfusionCMR as a reference. Second, the radiation dose incurred duringstress CCTA was relatively high. A 10-s scan duration was usedto determine the optimal enhancement time for dynamic stressCCTA. Therefore, a shorter dynamic stress CCTA scan durationshould be adopted to reduce the radiation dose.

Third, the TAG is influenced by the scanner hardware and the measurement technique. There is no standardised measurement method, and obtaining TAG remains time consuming. Although a semi-automated programme has been introduced, currently available software requires manual correction near branch vessels. Further refinements to the software are expected to reduce the time burden and facilitate applications in daily practice.

Fourth, we did not measure the TAG cut-off value in our study; we considered that a TAG cut-off value of –15.1 HU/10 mm, as previously described, would be significant.7 This value could have been influenced by the use of iodine contrast material, the CT protocol, or TAG calculation method. However, the value of –15.1 HU/10 mm is generally accepted, and therefore our data do not conflict with the results of other studies.

## Conclusion

This study found that the TAG of stress CCTA using a widearea detector CT yielded a high diagnostic performance as well as high sensitivity and specificity for the detection of haemodynamically significant coronary artery stenosis when compared with stress perfusion CMR. We believe that the addition of TAG to CCTA could allow for comprehensive anatomical and functional assessments of CAD, but this remains to be proven in appropriately designed prospective trials.
